# The declining interest in an academic career

**DOI:** 10.1371/journal.pone.0184130

**Published:** 2017-09-18

**Authors:** Michael Roach, Henry Sauermann

**Affiliations:** 1 Dyson School of Applied Economics and Management, Cornell University, Ithaca, New York, United States of America; 2 ESMT European School of Management and Technology, Berlin, Germany; 3 National Bureau of Economic Research, Cambridge, Massachusetts, United States of America; Iowa State University, UNITED STATES

## Abstract

There is increasing evidence that science & engineering PhD students lose interest in an academic career over the course of graduate training. It is not clear, however, whether this decline reflects students being discouraged from pursuing an academic career by the challenges of obtaining a faculty job or whether it reflects more fundamental changes in students’ career goals for reasons other than the academic labor market. We examine this question using a longitudinal survey that follows a cohort of PhD students from 39 U.S. research universities over the course of graduate training to document changes in career preferences and to explore potential drivers of such changes. We report two main results. First, although the vast majority of students start the PhD interested in an academic research career, over time 55% of all students remain interested while 25% lose interest entirely. In addition, 15% of all students were never interested in an academic career during their PhD program, while 5% become more interested. Thus, the declining interest in an academic career is not a general phenomenon across all PhD students, but rather reflects a divergence between those students who remain highly interested in an academic career and other students who are no longer interested in one. Second, we show that the decline we observe is not driven by expectations of academic job availability, nor by related factors such as postdoctoral requirements or the availability of research funding. Instead, the decline appears partly due to the misalignment between students’ changing preferences for specific job attributes on the one hand, and the nature of the academic research career itself on the other. Changes in students’ perceptions of their own research ability also play a role, while publications do not. We discuss implications for scientific labor markets, PhD career development programs, and science policy.

## Introduction

**Fig 1:**
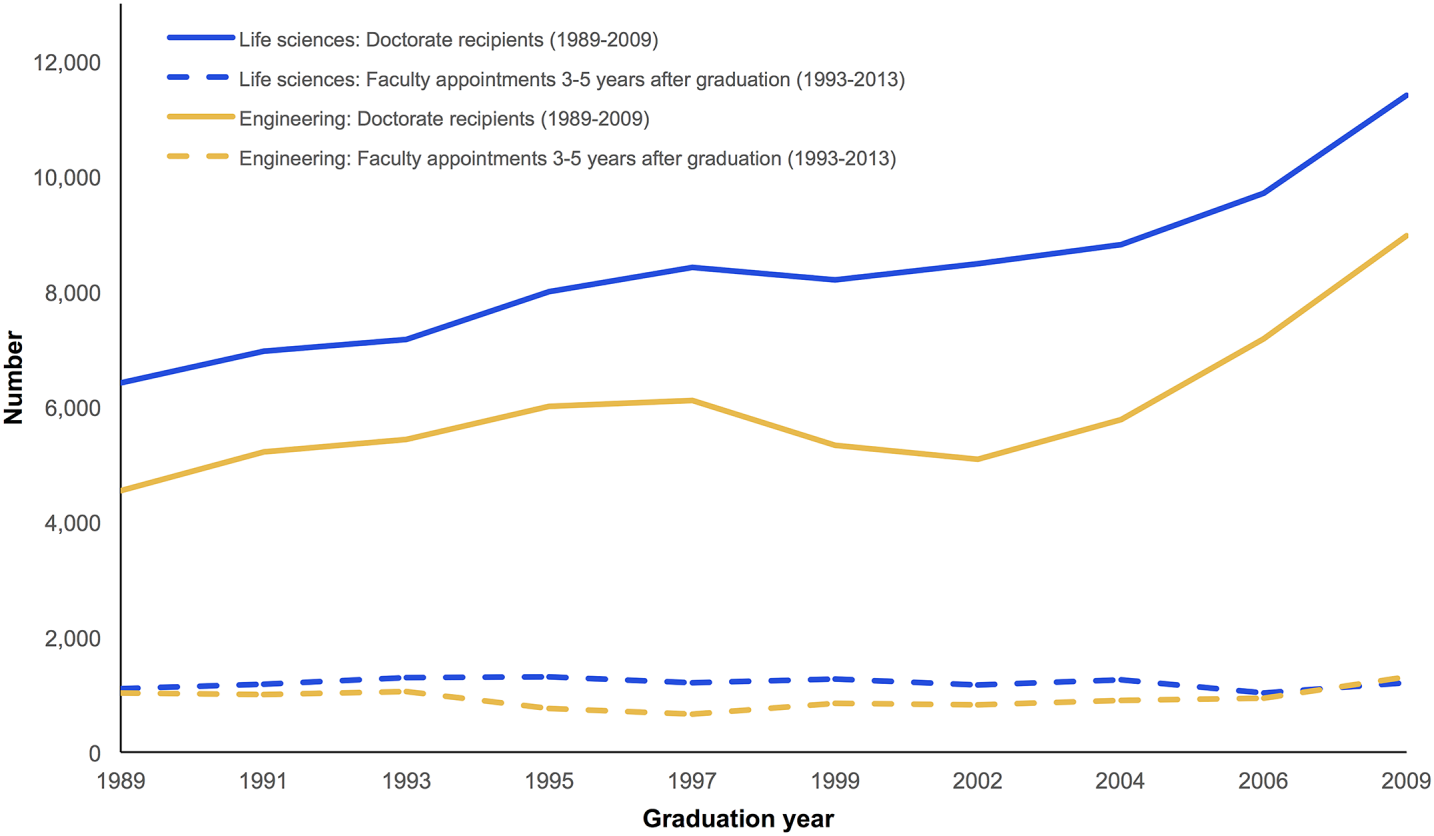
U.S. trends in life science & engineering doctorates and faculty appointments. Number of doctorate recipients and number of tenure-track faculty appointments 3–5 years after graduation (Data Source: NSF Survey of Doctorate Recipients; number of tenure-track faculty appointments calculated by the authors).

The number of science and engineering PhD degrees awarded in the U.S. has increased significantly over the last two decades (Fig 1). At the same time, the share of graduates holding tenure-track academic positions has declined, with the majority of science and engineering PhDs eventually taking positions outside of academia [1]. These trends have given rise to concerns that imbalances between the increasing supply of graduates and the limited number of available faculty positions may force many PhDs away from careers in academia [1–3]. On the other hand, recent research shows that many PhDs prefer non-academic careers upon graduation [4, 5], suggesting that labor market imbalances may not be as large as feared. However, it remains unknown whether the declining interest in an academic career is driven primarily by limited faculty job availability or whether it might also reflect substantive changes in career preferences irrespective of labor market conditions.

Using unique panel data from a survey of U.S. PhD students in science and engineering, this paper investigates how and why academic career preferences change over time during graduate training. Unlike prior studies that compare cohorts of students in the cross-section [4, 5], we observe the same PhD students first early in their program and then again three years later, allowing us to distinguish between students who remain interested in an academic career over time and those who lose interest. Moreover, we employ a unique measure that captures students’ career preferences independent from their labor market expectations, thus disentangling their “true” preference for an academic career from how difficult they think it will be to get an academic position. This measure allows us to provide clearer insights into students’ career preferences and the supply side of STEM labor markets.

We report two main results. First, the decline in Ph.D. students’ interest in an academic career is not a general phenomenon across all students, but rather is a significant divergence between students who remain highly interested in an academic career and others who lose interest in an academic career entirely. Second, we show that the decline we observe is driven not by expectations about the academic job market, but instead partly reflects changes in students’ preferences for specific aspects of the faculty career, such as performing basic research and having freedom to choose research projects.

Although labor market conditions almost certainly prevent some doctoral students who remain interested in an academic career from obtaining a faculty position, our findings suggest that many students turn away from academia for reasons other than the lack of faculty positions. As such, discussions of PhD students’ career goals and career pathways should consider a broad set of market and non-market factors. Our findings also provide urgency to the National Academies’ recent call for better data on students’ career preferences [6], and we present a measure that may be useful in such data collection efforts. Our results suggest the need for greater flexibility in graduate programs and may help faculty advisors, program administrators, and policy makers to improve STEM training experiences. Our findings also have important implications for research on STEM labor markets, universities’ efforts to improve graduate education, and federal efforts to track and manage the STEM labor supply.

## Background

Before we examine changes in students’ academic interests empirically, it is useful to consider briefly some of the potential reasons for such changes. While this discussion is far from exhaustive, it is meant to introduce some of the market and non-market factors that may be at play. To begin, a common explanation is that PhD students are discouraged from pursuing an academic career because they learn about the limited number of faculty openings and the low likelihood of obtaining a tenure-track position [2, 7, 8]. As such, PhD students’ “true” preference for an academic career may not have changed, but their expectations of being able to obtain a faculty position have. To the extent that stated career preferences are influenced by labor market expectations, they would understate the share of graduates who aspire to an academic career.

Students may also lose interest in an academic career for reasons unrelated to labor market conditions. For example, during the course of the PhD program, students may gain deeper insight into the life of a faculty member and realize that this career is not what they expected [9, 10]. Although common stereotypes highlight attractive features such as autonomy, the opportunity to do curiosity-driven research, and inspiring social interactions in an invisible college of peers, the faculty career is not without challenges. For example, funding conditions have deteriorated in many fields and junior faculty in particular face significant difficulties in securing grants to fund their work [7]. As such, faculty members spend significant amounts of time on acquiring and administering resources, which detracts from the time they can spend on research [11]. Moreover, both funding agencies and tenure committees place great emphasis on quantitative measures of research output, increasing the pressure to generate publications and sometimes detracting from curiosity driven discovery [7]. Students may also realize that for faculty members, “doing research” does not always mean hands-on investigation but often involves administrative tasks in managing a lab and conveying research to external audiences [12, 13]. Finally, while autonomy is often highlighted as one of the key benefits of being an academic, success in such an unstructured occupation requires the ability to balance competing demands from teaching, research, and administration. It also requires the willingness to take initiative, the ability to make tough choices regarding which projects to pursue, and good sense for when to persist or give up on a project that seems likely to fail [12, 14].

Although these and other challenges associated with being a faculty member have been highlighted in prior scholarly work and policy discussions, many applicants do not think explicitly about career options when enrolling in a PhD program [15, 16]. Moreover, it is unlikely that the various facets of the faculty career can be understood simply by reading about them: Students are likely to realize what it means to be a faculty member primarily through extended exposure to mentors and peers during graduate training, and through their own involvement in research and teaching [10, 17]. As such, students may gradually re-evaluate the attractiveness of the faculty career over the course of the PhD program or may realize that their own interests are not a fit for this career path. Upon experiencing the highly competitive nature of academia and gaining a better understanding of their own abilities, students may also re-evaluate their chances of success, or the time and effort they would have to commit in order to succeed. Of course, training experiences are not uniform [17], and while some students may realize that the faculty career is not the best fit for them, others may remain highly interested and some may even increase their commitment to this career path.

## Materials and methods

We examine changes in PhD students’ career interests using a longitudinal survey that followed 854 students over the course of their PhD training in the life sciences (36%), chemistry (12%), physics (18%), engineering (24%), and computer science (10%). Unlike prior studies that compare cohorts of students in the cross-section [4, 5], our longitudinal approach allows us to directly assess changes for a given person and to distinguish between PhD students who remain interested in an academic career and those who lose interest during graduate training. To obtain the initial sample, we identified 39 tier-one U.S. research universities with doctoral programs in science and engineering fields by consulting the National Science Foundation’s reports on earned doctorates [18]. Our selection of universities was based primarily on program size while also ensuring variation in private/public status and geographic region. The 39 universities in our sample produced roughly 40% of the graduating PhDs in science and engineering fields in 2009 [2, 7, 8]. The questionnaire was validated by inviting a select sample of PhD students at the investigators’ universities to complete the survey followed by an exit interview to probe students’ understanding of key questions and to solicit feedback on the instrument. The respective Institutional Review Boards at Cornell University and the Georgia Institute of Technology approved this survey. Participation in the survey was voluntary and subjects consented by completing the survey.

Respondents were contacted through email addresses obtained from university department and research lab websites and invited to participate in an online survey regarding their PhD experience and career goals. The first survey was administered in February 2010 to nearly 30,000 PhD students and postdocs at various stages of their training, with a response rate of 30%. As part of the 2010 survey, we asked respondents to provide us with a permanent email address (e.g., a Gmail account), which was used to contact respondents in February 2013 with a follow-up questionnaire. If respondents did not provide an email in the 2010 survey (20% of respondents), we used the original university email address from the 2010 survey. In this study we focus on the subset of 854 respondents who were first or second year PhD students in 2010 and who responded as fourth or fifth year students in 2013, with a 40% response rate for the second survey.

To examine potential nonresponse bias in this sample, we regressed response status in 2013 on key characteristics from the 2010 survey. We find that the likelihood of a response to the follow up was higher for respondents who were US citizens and who were in the second (vs. first) year of their PhD studies. Controlling for these factors, we do not find significant differences with respect to career interests. We include the relevant variables as controls in our regression analyses, which are described in detail in the Results section below. S1 Table reports summary statistics. The specific survey questions used in this study are reported in S1 Text.

## Results

Our empirical analysis involves three parts. We first document changes in career preferences over time using longitudinal data and explore whether changes are a general phenomenon or are limited to certain parts of the population. We then examine whether the changes we observe may be driven by students’ expectations regarding labor market conditions using nonparametric methods and also explore other potential reasons for changes in career preferences including changes in interests in different types of tasks or job attributes and changes in subjective ability. Finally, we present a series of regression analyses that allow us to examine the potential drivers of changes in career preferences jointly while controlling for demographic characteristics and other factors.

### Assessing changes in academic career interests

We rely on direct measures of career interests rather than interpreting observed career transitions that may confound both preferences and labor market constraints [19, 20]. We asked respondents at both points in time: “Putting job availability aside, how attractive or unattractive do you personally find each of the following careers?” Although the survey asked about a range of research and non-research careers inside and outside academia, this paper focuses on students’ interest in “university faculty with an emphasis on research or development” (*academic career*). Respondents rated this career independently from other careers using a 5-point scale ranging from “extremely unattractive” (1) to “neither attractive nor unattractive” (3) to “extremely attractive” (5). By explicitly asking respondents to disregard current labor market conditions, our measure attempts to capture PhD students’ interest in an academic career independent of factors that may hinder their ability to obtain an academic career, such as a limited number of available faculty positions.

**Fig 2:**
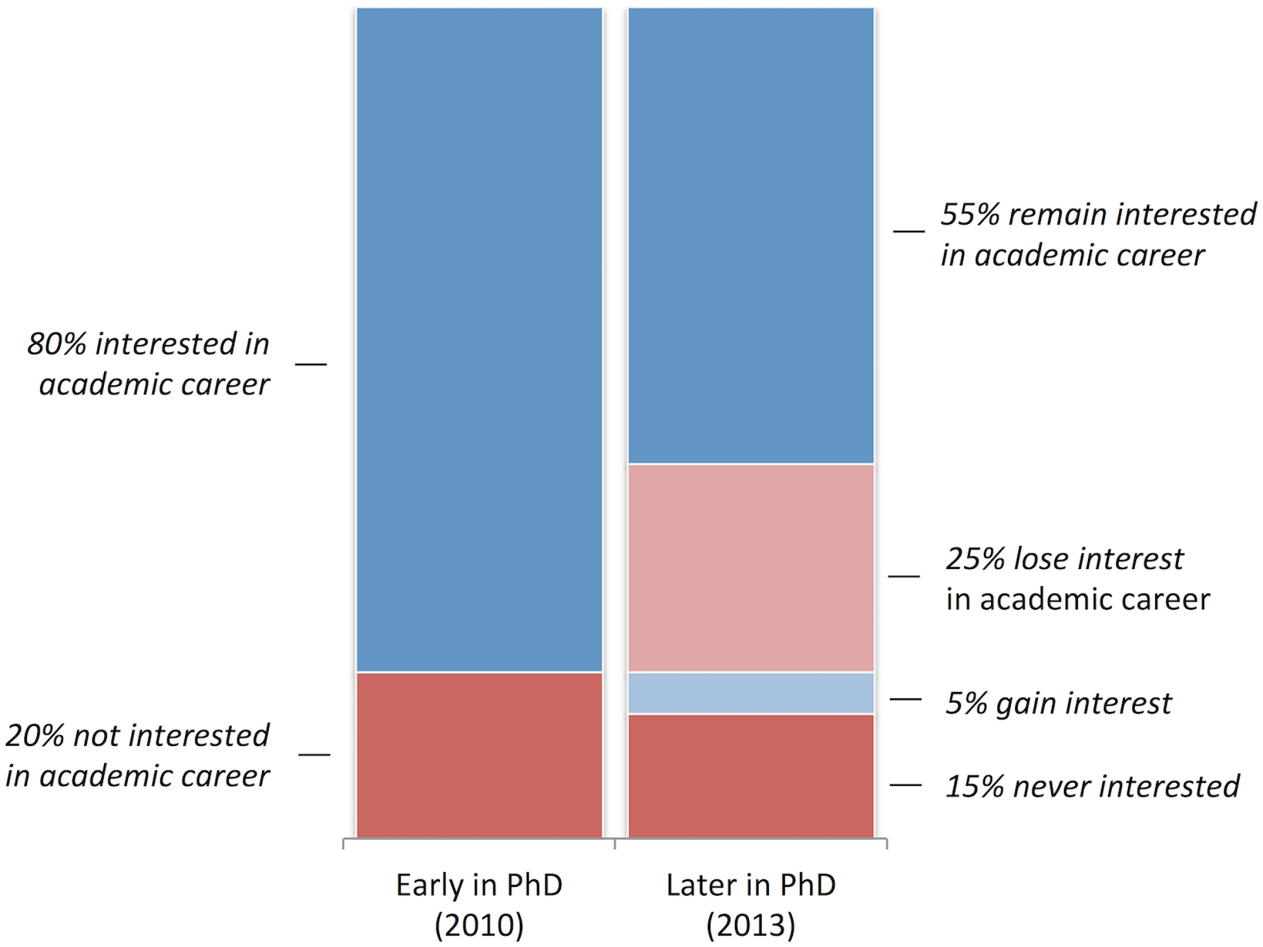
Change in academic career interests during the PhD program.

We dichotomized the scale to distinguish between PhD students who are interested in an academic research career (i.e., ratings of “extremely attractive” or “attractive”) and those who are not (i.e., ratings of “neither attractive nor unattractive”, “unattractive”, or “extremely unattractive”) early in the PhD program (2010), as illustrated in Fig 2. We similarly coded students’ interest in an academic research career three years later (2013) when they were in an advanced stage of their PhD and near graduation [21]. To construct our change measure, we then code students who are interested in an academic research career in both periods as remaining interested and students who were interested early in the PhD but are no longer interested later in the program as losing interest in an academic career.

Although the preponderance of all PhD students (80%) started the program with an interest in an academic career, three years later just over half (55%) remain interested in an academic career and one-quarter (25%) lose interest. Put differently, nearly one-third of doctoral students who started the PhD program interested in an academic research career lost interest in that career by the time they neared graduation. Moreover, PhD students who lose interest in an academic career show a substantial decline in their ratings on the original 5-point attractiveness scale, dropping from a mean of 4.3 in 2010 to 2.2 in 2013, with two-thirds of them now reporting that an academic career is either “unattractive” or “extremely unattractive”. As we would expect given the construction of our measure, the average attractiveness score does not change significantly among PhD students who remained interested in an academic career (mean of 4.5 in 2010 and 4.4 in 2013). Thus, the declining interest in an academic career is not a general phenomenon, but rather reflects a significant divergence between PhDs who remain interested in an academic career and others who lose interest in academia entirely.

Approximately 20% of all PhD students started the program uninterested in an academic career, and over time 15% remain uninterested and 5% gain interest. Table 1 reports the change in academic career interests across broad fields of science and engineering. Due to limited sample size, our main analysis uses the pooled sample, with controls for 36 subfields in regression models. We report auxiliary analyses for selected fields towards the end of the paper.

**Table 1:** Academic career interests by field. Levels early in the PhD (2010) and changes from 2010 to 2013.

Field of Study	Obs.	*2010*	*Change from 2010 to 2013*			
		Academic career interest early in PhD	Remain interested	Lose interest	Gain interest	Never interested
Life sciences	313	83%	59%	25%	4%	12%
Chemistry	107	60%	30%	30%	8%	32%
Physics	154	92%	66%	26%	4%	5%
Engineering	193	76%	52%	24%	6%	18%
Computer science	87	80%	65%	15%	8%	12%
**All Fields**	**854**	**80%**	**55%**	**25%**	**5%**	**15%**

Table 2 examines potential differences by demographic characteristics. A greater share of men start the PhD with an interest in an academic career relative to women (83% vs. 75%), and this difference is highly significant (t-statistic -2.99, p-value 0.003). Moreover, this difference persists over time with 59% of men remaining interested in an academic career compared to 50% of women. Although levels of career interests differ by gender, similar shares of men and women report a decline in their interest in an academic career over time (24% and 25%, respectively); 19% of women were not interested in academic research in either time period compared to 12% of men. These results are broadly consistent with prior cross-sectional evidence [22].

**Table 2:** Academic career interests by gender and nationality.

	Obs.	*2010*	*Change from 2010 to 2013*			
		Academic career interest early in PhD	Remain interested	Lose interest	Gain interest	Never interested
**Gender**						
Men	500	83%	59%	24%	5%	12%
Women	345	75%	50%	25%	6%	19%
**Nationality**						
U.S. citizens	626	79%	51%	27%	6%	16%
Non-U.S. citizens	219	84%	68%	16%	5%	11%

The difference in the share of U.S. citizens (79%) and foreign PhD students (84%) interested in an academic career at the beginning of the PhD is only marginally significant (t-statistic 1.73, p-value 0.08). However, 27% of U.S. citizens lose interest in an academic career compared to only 16% of foreign PhD students. As they near graduation, 51% of U.S. citizens remain interested in an academic career compared to 68% of foreign students, and this difference is highly significant (t-statistic 4.38, p-value 0.001). Although these patterns are intriguing, a detailed examination of these differences is beyond the scope of this paper.

### Non-parametric analyses of potential reasons for changes in academic interests

We now examine whether the changes in academic career interests observed above are associated with students’ expectations of labor market conditions using nonparametric methods. We also examine the extent to which the declining interest in an academic career is associated with changes in preferences for work activities and job attributes, as well as proxies for students’ ability. (See S1 Table for a comprehensive list of variables).

#### Labor market expectations

**Fig 3:**
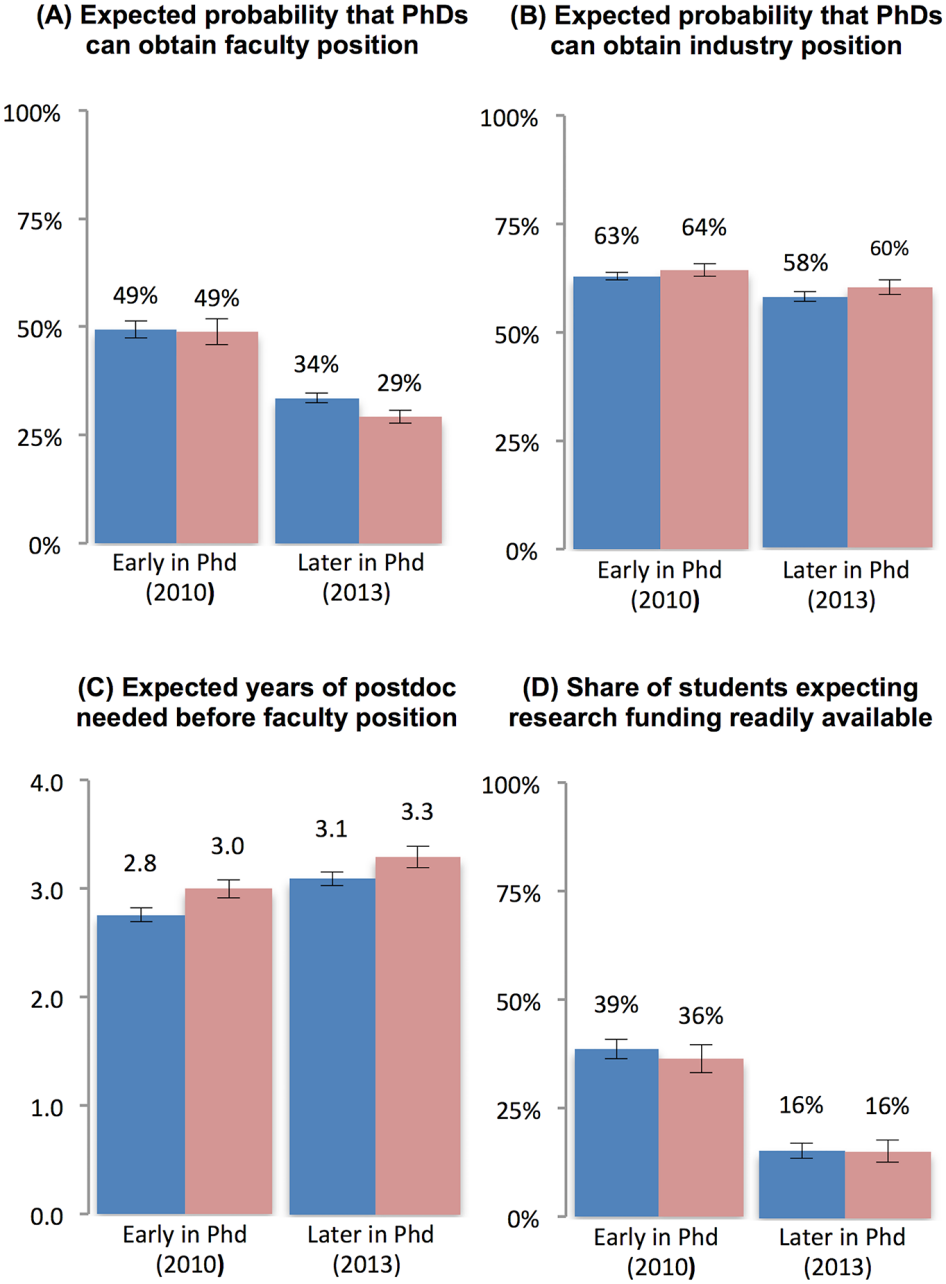
Changes in expectations of academic labor market conditions. Individuals who remain interested in an academic career drawn in dark blue and those who lose interest in an academic career in light red; (A) expected probability that a PhD in their field can obtain a faculty position after graduation; (B) expected probability that a PhD in their field can obtain an industrial R&D position after graduation; (C) expected number of years of postdoctoral research needed to obtain a faculty position; (D) expected availability of funding for academic research.

As noted in the description of the measure of career interests, the survey question was designed to capture career preferences independent from labor market conditions. To validate this important aspect of our approach, we now examine the relationships between academic career interests and three factors related to the academic labor market as illustrated in Fig 3 and summarized in Tables 3 & 4.

**Table 3:** Means of key variables by stage in the PhD program.

	*Early in PhD (2010)*				*Later in PhD (2013)*				*Change during PhD (2010 to 2013)*			
	Remain interested	Lose interest	t-test	p-value	Remain interested	Lose interest	t-test	p-value	Remain interested	Lose interest	t-test	p-value
**Labor market expectations**												
Expected probability of faculty position	49%	49%	0.29	0.77	34%	29%	2.31	0.02	-16%	-20%	1.66	0.10
Expected probability of industrial R&D position	63%	64%	0.82	0.41	58%	60%	1.13	0.26	-4%	-5%	0.41	0.68
Years of postdoc required for faculty position	2.76	2.97	1.87	0.06	3.11	3.28	1.54	0.12	0.31	0.30	-0.15	0.88
Availability of research funding	38%	36%	0.56	0.58	15%	15%	0.09	0.93	-23%	-21%	-0.43	0.67
**Work preferences**												
Interest in basic research	92%	87%	2.49	0.01	92%	53%	13.93	0.00	0%	-33%	9.55	0.00
Interest in applied research	93%	94%	-0.29	0.77	93%	84%	3.58	0.00	0%	-10%	3.25	0.00
Interest in commercialization	42%	44%	-0.38	0.70	38%	55%	-4.19	0.00	-4%	11%	-3.40	0.00
Importance of salary	79%	78%	0.13	0.89	80%	80%	0.08	0.94	2%	2%	-0.05	0.96
Importance of freedom	92%	86%	2.51	0.01	88%	61%	8.46	0.00	-4%	-25%	5.72	0.00
**Ability**												
Self-perceived ability	6.39	6.00	3.21	0.00	6.98	5.99	8.15	0.00	0.59	-0.01	4.21	0.00
Number of publications	0.96	0.80	1.37	0.17	2.72	2.29	2.32	0.02	1.87	1.66	1.33	0.18

**Table 4:** Means of key variables by change in academic career interests.

	*Remain interested*				*Lose interest*			
	Early in PhD (2010)	Later in PhD (2013)	t-test	p-value	Early in PhD (2010)	Later in PhD (2013)	t-test	p-value
**Labor market expectations**								
Expected probability of faculty position	49%	34%	12.46	0.00	49%	29%	10.42	0.00
Expected probability of industrial R&D position	63%	58%	4.43	0.00	64%	60%	2.55	0.01
Years of postdoc required for faculty position	2.76	3.11	-4.59	0.00	2.97	3.28	-2.76	0.01
Availability of research funding	38%	15%	9.01	0.00	36%	15%	5.29	0.00
**Work preferences**								
Interest in basic research	92%	92%	0.42	0.67	87%	53%	8.68	0.00
Interest in applied research	93%	93%	0.14	0.89	94%	84%	3.76	0.00
Interest in commercialization	42%	38%	1.64	0.10	44%	55%	-2.88	0.00
Importance of salary	79%	80%	-0.78	0.43	78%	80%	-0.54	0.59
Importance of freedom	92%	88%	2.30	0.02	86%	61%	6.64	0.00
**Ability**								
Self-perceived ability	6.39	6.98	-7.72	0.00	6.00	5.99	-0.06	0.95
Number of publications	0.96	2.72	-17.91	0.00	0.80	2.29	-10.81	0.00

First, we asked respondents both in early in their PhD (2010) and again three years later at an advanced stage of their PhD (2013): “What do you think is the probability that a PhD in your field can find the following positions after graduation (and any potential postdocs)”, where the listed positions included “university faculty with an emphasis on research or development” as well as “established firm job with an emphasis on research or development.” Respondents reported expected probabilities on a scale ranging from 0–100%. Panel A in Fig 3 shows that early in the program both groups expect that nearly half of PhD graduates in their field can obtain a faculty position at some point in their career. Over time these expectations decrease significantly for all students, irrespective of whether they remain interested in an academic career or lose interest. Although students who lose interest have significantly lower expectations later in the PhD program regarding the probability of obtaining a faculty job than those who remain interested (34% vs. 29%, p = 0.02), the change in expectations is similar in magnitude and not statistically different between the two groups (Table 3, -20% change vs. -16% change, p = 0.10). Panel B in Fig 3 shows that students who remain interested in an academic career and those who lose interest report similar expected probabilities of obtaining an industrial R&D position, and this probability decreases only slightly over time.

Second, a lower availability of tenure-track positions is likely reflected in a longer duration of postdoctoral appointments before graduates can find a tenure-track position [3, 6, 15]. Accordingly, we asked PhDs “How many years of postdoc experience do you think are required on average to obtain a university faculty position with an emphasis on research or development in your field?” Respondents answered on a multiple-choice scale that ranged from 0 years (i.e., no postdoc required) to 5 or more years. Panel C in Fig 3 shows that students’ expectations regarding the duration of postdoctoral training required increased slightly over the course of the PhD program, consistent with an increasing awareness of labor market challenges. However, we find no significant differences in the changes in expectations between students who lose interest in faculty careers and those who remain interested (increase of 0.30 and 0.31 years, respectively, Table 3).

Finally, we consider whether increasing awareness of the challenges of obtaining research funding might explain the declining interest in an academic career. We asked students in both periods “To what extent do you think research funding is available to faculty members at a research university?” using a 5-point scale ranging from 1 (“extremely low”) to 5 (“extremely high”). We dichotomized responses to distinguish students who believed that research funding was readily available (“extremely high” and “high”) and those who did not. Panel D in Fig 3 shows that the share of PhD students with expectations that research funding is readily available declined significantly for both groups over the course of the PhD program, and the decline is not significantly larger among student who lose interest in an academic research career (Table 3).

Taken together, these results show that while PhD students adjust their expectations of labor market conditions over time, an increasing awareness of labor market challenges is shared by students who remain interested in an academic research career and those who lose interest. As such, differences in the degree to which labor market expectations changed are unlikely to explain why some students lose interest in the academic career while others remain highly interested. Note that even if changes in labor market expectations are similar for both groups, it could be that these changes had a larger impact on one group of students than the other. We explore this possibility below but find no evidence that this was the case.

#### Preferences for work activities and job attributes

We now turn to potential non-market reasons for changes in students’ academic interests. We first explore the possibility that students may lose interest in the faculty career because of changes in their preferences for different types of work activities such as basic research or for job attributes such as freedom and pay. Such preferences for work activities and job attributes have been shown to predict career choice [23, 24], but we are not aware of studies using a dynamic perspective to examine changes in these preferences and changes in career interests.

We asked in both waves of the survey: “When thinking about the future, how interesting would you find the following kinds of work?”, using a 5-point scale ranging from “extremely uninteresting” to “extremely interesting.” Work activities included basic research (“research that contributes fundamental insights or theories”), applied research (“research that creates knowledge to solve practical problems”) and commercialization (“commercializing research results into products or services”). To measure preferences for job attributes students were asked “When thinking about an ideal job, how important is each of the following factors to you?”, using a 5-point scale ranging from “not at all important” to “extremely important.” The listed factors include “financial income (e.g., salary, bonus, etc.)” and “freedom to choose research projects”.

**Fig 4:**
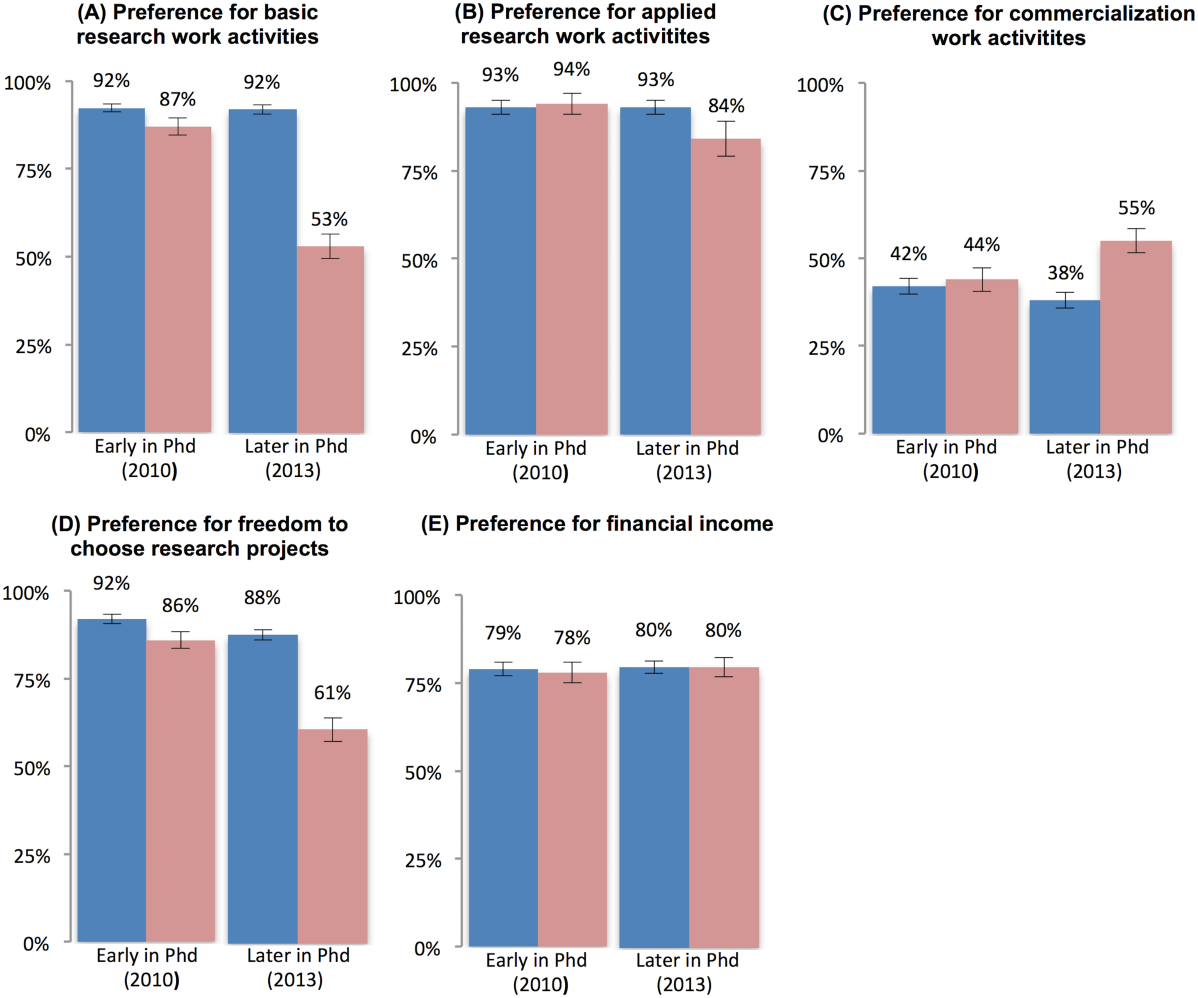
Changes in preferences for work activities and job attributes. Individuals who remain interested in an academic career are drawn in dark blue and those who lose interest in an academic career in light red; (A) preference for engaging in basic research work activities; (B) preference for engaging in applied research work activities; (C) preference for engaging in commercialization work activities; (D) preference for freedom in choosing work projects; (E) preference for financial income.

To simplify comparisons, we dichotomize these measures and distinguish between students who report strong preferences for the different work activities (“interesting” or “extremely interesting”) or job attributes (“important” or “extremely important”) and those who report indifferent or weak preferences. Fig 4 shows that early in the PhD program the vast majority of PhDs have a strong preference for basic and applied research, as well as for freedom in choosing research projects. PhD students who remained interested in an academic career later in the PhD changed little over time with respect to these preferences. Among students who lost interest, however, the share with strong preferences for basic research, applied research, and freedom decreased significantly (Table 3), while the share with a strong preference for commercialization increased. There is no significant difference between groups and no significant change over time in preferences for financial income (Table 3).

Taken together, our observations are consistent with the notion that preferences for work activities and job attributes shape students’ career interests [23, 25] and suggest that the decreased interest in a faculty career partly reflects changes in students’ preferences for certain aspects of this career path such as the focus on basic research. We note, however, that these data do not allow for a clear identification of causality. While the longitudinal nature of the analysis reduces concerns about omitted variables (e.g., a comparison of changes eliminates the influence of fixed individual characteristics), we cannot rule out reverse causality, i.e., that changes in career interests may lead to changes in preferences for work activities and job attributes.

#### Ability

Over the course of their graduate studies, PhD students are likely to also gain a better understanding of their own ability. Students who realize that they are not at the top of the ability distribution or who are less successful in developing publishable research than others may understand that it will be difficult to succeed in the highly competitive academic research enterprise, even if they were able to secure a faculty position. To examine whether learning about ability may explain changes in career preferences, we use two different proxies for ability. First, we asked respondents in both waves of the survey: “How would you rate your research ability relative to your peers in your area of specialization?”, using a sliding scale ranging from 0 (among the least skilled) to 5 (average) to 10 (among the most skilled). This measure has a mean of 6.17 early in the PhD and 6.64 later in the PhD, suggesting that students feel that their (relative) ability increases slightly with time in the program. To obtain a more objective proxy for ability, we also asked respondents to indicate how many published or accepted articles in peer-reviewed journals listed them as authors. As expected, this measure increases sharply over the course of the PhD training, rising from a mean of 0.87 publications early in the PhD to 2.52 publications three years later. Subjective and objective measures are significantly correlated in both time periods, although these correlations are only of moderate size (0.18 in 2010 and 0.21 in 2013).

**Fig 5:**
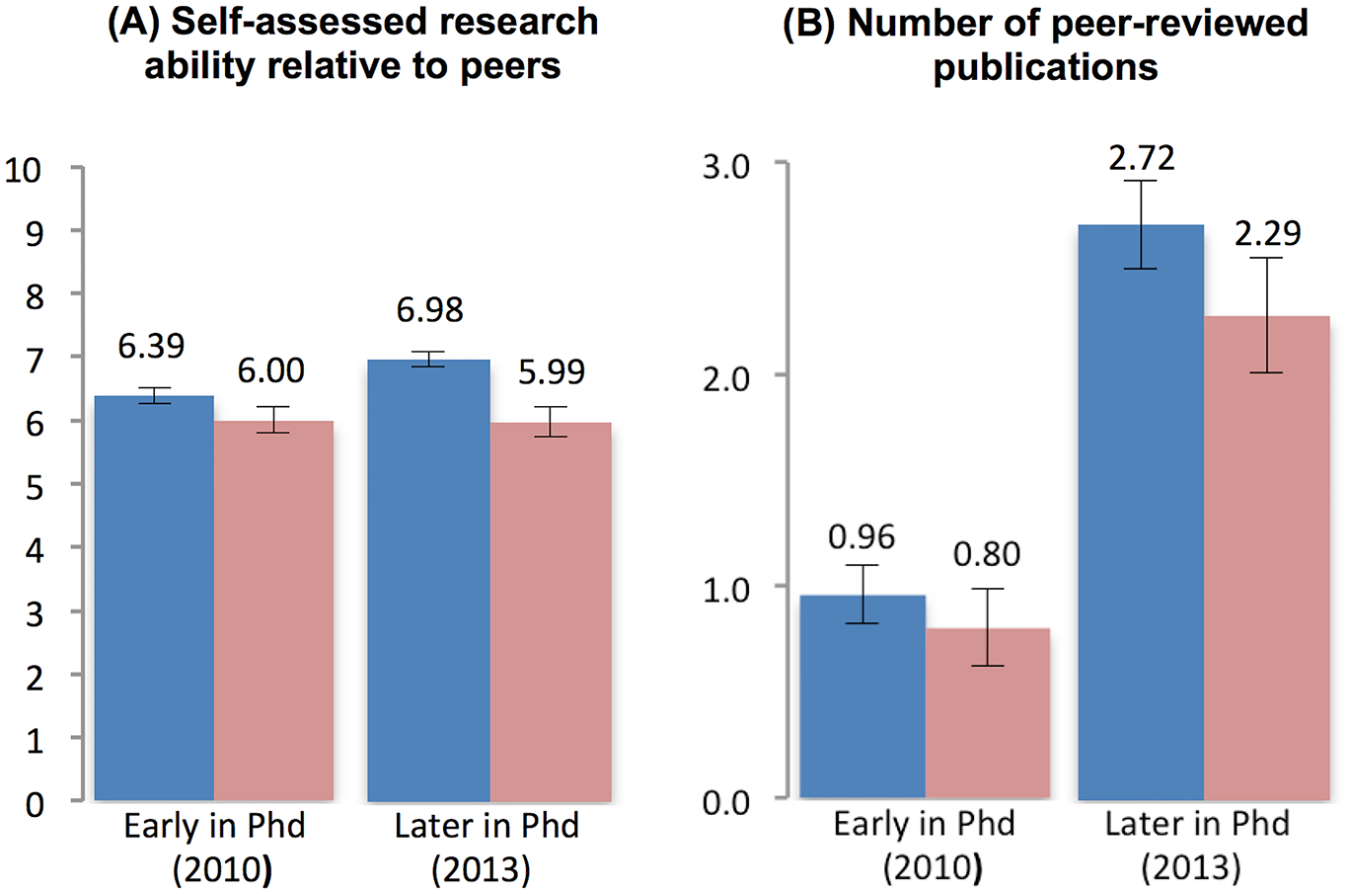
Changes in ability. Individuals who remain interested in an academic career are drawn in dark blue and those who lose interest in an academic career in light red; (A) self-assessed research ability relative to peers in their field: (B) number of academic articles published or accepted for publication.

Fig 5 shows that students who remain interested in the faculty career start with higher levels of subjective ability (6.39 vs. 6.00, t-statistic = 3.21 p-value = 0.001, Table 3) and publications (0.96 vs. 0.80, t-statistic = 1.37 p-value = 0.170, Table 3) than those who lose interest. More importantly, subjective ability increases significantly among those who remain interested in academia (from 6.39 to 6.97; t-statistic = 7.72 p-value = 0.001, Table 4), while it remains unchanged among those who lose interest in academia (t-statistic = 0.06 p-value = 0.951, Table 4). Publication counts increases for both groups, but only slightly more for PhD students who remain interested in academia (increase by 1.7 publications) than for those who lose interest (increase by 1.5).

Taken together, we find evidence that changes in career interests may partly reflect changes in students’ assessments of their own ability and performance. But again, the observed correlations do not imply causation. In particular, we cannot rule out that students who decide not to pursue a faculty position are less driven to publish their research. This concern is somewhat mitigated by the observation that publications also have considerable value when students seek non-academic jobs and that publishing decisions in academic labs are to a large extent driven by the strong career incentives of advisors [1, 26, 27].

### Regression analyses

#### Main models

We now examine these relationships systematically through a series of regression analyses that allow us to examine more carefully two different issues. First, they allow us to correlate changes in career preferences with changes in independent variables such as labor market expectations or ability, similar to the approach used in the nonparametric analysis. Towards this end, we estimate a multinomial regression model that uses as the dependent variable a categorical variable distinguishing PhD students who remain interested in an academic career (base category of the dependent variable), PhD students who lose interest, PhD students who gain interest, and PhD students who were never interested in an academic career. Independent variables include changes in labor market expectations, preferences for job attributes, and ability, as well as a range of control variables such as field of study, the National Research Council ranking of the students’ primary department [28], and demographic characteristics such as gender and citizenship (see S1 Table for key variables). The basic structure of this regression is:

$$\text{CHG}\_\text{ACAD}\_{\text{CAREER}}_{i}={\text{β}}_{1}\text{CHG}\_{\text{MARKET}}_{i}+{\text{β}}_{2}\text{CHG}\_{\text{PREFS}}_{i}+{\text{β}}_{3}\text{CHG}\_{\text{ABILITY}}_{i}+{\text{β}}_{4}\text{CHG}\_{\text{CONTROLS}}_{i}+{\text{ β}}_{5}{\text{CONTROLS}}_{i}+{\text{ε}}_{I},$$ (1)

where CHG_ACAD_CAREER_*i*_ is a categorical variable classifying respondent *i* by whether and how the interest in academic research has changed, CHG_MARKET_*i*_ is a vector of variables capturing changes in the respondent’s market expectations, CHG_PREFS_*i*_ is a vector of variables capturing changes in preferences for work activities and job attributes, CHG_ABILITY_*i*_ is a vector of changes in proxies for ability, CHG_CONTROLS_*i*_ is a vector of changes in time-varying controls, and CONTROLS_*i*_ is a vector of time invariant controls. By using changes for both the dependent and key independent variables, this model also partly addresses concerns about otherwise unobserved time-invariant heterogeneity across individuals, including potential biases in survey response behavior.

Considering changes in independent variables as predictors of changes in the outcome of interest over time is based on the premise that levels of independent variables predict levels of outcomes at a given point in time. For example, if a decrease in students’ interest in basic research between 2010 and 2013 explains a decrease in the attractiveness of a faculty career, then we would also expect that at a given point in time, students with a weaker interest in basic research report the faculty careers as less attractive. As such, we estimate a second set of regressions using cross-sectional data from each wave of the survey. In addition to showing which factors are correlated with academic career interests at a given point in time, these regressions allow us to examine whether key coefficients change between 2010 and 2013, e.g., whether the interest in basic research has become a more or less important predictor of the attractiveness of a faculty career. The basic structure of these regressions (estimated using ordered logit) is:

$$\text{ACAD}\_{\text{CAREER}}_{it}={\text{β}}_{1}{\text{MARKET}}_{it}+{\text{β}}_{2}{\text{PREFS}}_{it}+{\text{β}}_{3}{\text{ABILITY}}_{it}+{\text{β}}_{4}{\text{CONTROLS}}_{it}+{\text{ε}}_{I},$$ (2)

where ACAD_CAREER_*it*_ is the respondent’s rating of interest in the faculty research career, and where the subscript t stands for either 2010 or 2013.

Taken together, the two sets of regressions provide insights into the degree to which the decline in academic career interests may be explained by changes in the levels of important predictor variables (e.g., labor market expectations, individual preferences or perceived ability), but also by changes in the role these variables play in shaping career interests at a given point in time (see [29]).

Table 5 presents multinomial regression coefficients as relative risk ratios such that coefficients >1 indicate a positive relationship, coefficients = 1 indicate no relationship, and coefficients <1 indicate a negative relationship. Model 1 includes only control variables. Model 2 adds measures of changes in labor market expectations, which are consistent our earlier non-parametric finding that changes in labor market expectations have no systematic relationship with changes in respondents’ interest in the academic career. Also consistent with the nonparametric analysis, Model 3a shows that respondents whose interest in basic research has decreased are significantly more likely to lose interest in an academic career relative to remaining interested (the omitted category of the dependent variable). We also observe a marginally significant association between an increasing interest in commercialization activities and losing interest in an academic career (p-value = 0.075). Students whose preference for research freedom has decreased are also more likely to lose interest in an academic career, while respondents who feel that their research ability has increased are less like to lose interest [30]. Although our focus is on students who lose interest in academia rather than those who gain interest, Model 3b shows that PhD students who gain interest in an academic career also exhibit a significantly decreased interest in commercialization, an increased preference for research freedom and increased subjective ability, reinforcing the importance of these variables in explaining changes in academic career interests.

**Table 5:** Multinomial regressions predicting change in academic career interest (categorical DV).

*Method*	Multinomial logit								
*Description*	*Baseline*			*Labor market factors*			*Market & non-market factors*		
*Dependent variable (relative to remain interested in an academic career)*	Lose interest	Gain interest	Never interested	Lose interest	Gain interest	Never interested	Lose interest	Gain interest	Never interested
*Model*	(1a)	(1b)	(1c)	(2a)	(2b)	(2c)	(3a)	(3b)	(3c)
***Labor market expectations***									
Chg. availability of faculty positions				0.55	1.23	1.57	0.53	1.55	1.38
				[0.27,1.11]	[0.39,3.91]	[0.61,4.06]	[0.23,1.24]	[0.46,5.21]	[0.53,3.60]
Chg. availability of industry positions				1.71	2.19	1.66	2.00	2.25	2.07
				[0.76,3.84]	[0.31,15.27]	[0.72,3.80]	[0.77,5.22]	[0.35,14.40]	[0.79,5.45]
Chg. number of years of postdoc				0.99	1.00	1.04	0.97	0.96	1.02
				[0.87,1.12]	[0.77,1.31]	[0.83,1.31]	[0.83,1.14]	[0.71,1.28]	[0.81,1.28]
Chg. availability of research funding				0.92	0.91	0.97	0.95	0.92	0.94
				[0.79,1.09]	[0.65,1.28]	[0.81,1.18]	[0.79,1.13]	[0.64,1.32]	[0.77,1.15]
***Work preferences***									
Chg. basic research work activities							0.47***	0.97	0.80
							[0.37,0.61]	[0.62,1.53]	[0.58,1.10]
Chg. applied research work activities							0.80	1.21	0.78
							[0.62,1.03]	[0.58,2.53]	[0.55,1.11]
Chg. commercialization work activities							1.19	0.71*	0.91
							[0.98,1.43]	[0.52,0.98]	[0.74,1.13]
Chg. financial income							1.03	0.79	1.02
							[0.75,1.40]	[0.47,1.32]	[0.74,1.41]
Chg. freedom to choose projects							0.67***	1.68**	0.68*
							[0.53,0.85]	[1.16,2.44]	[0.50,0.93]
***Ability***									
Chg. self-perceived ability							0.83**	1.40**	1.10
							[0.72,0.94]	[1.10,1.77]	[0.94,1.28]
Chg. number of publications							0.95	0.98	0.80*
							[0.86,1.06]	[0.82,1.18]	[0.66,0.98]
***Control variables***									
Department NRC ranking (2010)	0.99	0.98	1.00	0.99	0.98	1.00	0.99	0.98	1.00
	[0.97,1.01]	[0.94,1.02]	[0.98,1.02]	[0.97,1.01]	[0.94,1.03]	[0.98,1.02]	[0.97,1.02]	[0.94,1.03]	[0.98,1.03]
Chg. thought about career	1.17	0.87	0.96	1.18	0.89	0.97	1.26*	0.84	1.01
	[0.98,1.40]	[0.64,1.18]	[0.76,1.21]	[0.99,1.40]	[0.65,1.21]	[0.76,1.24]	[1.05,1.52]	[0.54,1.31]	[0.78,1.30]
Male	0.91	0.53*	0.60*	0.89	0.50**	0.58*	0.74	0.44**	0.53**
	[0.54,1.51]	[0.32,0.86]	[0.39,0.91]	[0.54,1.47]	[0.31,0.81]	[0.37,0.88]	[0.41,1.34]	[0.26,0.77]	[0.33,0.85]
Chg. married	0.93	0.95	1.50	0.96	1.00	1.54*	0.99	0.92	1.51
	[0.66,1.30]	[0.54,1.67]	[1.00,2.26]	[0.69,1.35]	[0.55,1.85]	[1.02,2.32]	[0.66,1.48]	[0.48,1.74]	[0.99,2.31]
Chg. number of children	1.84	0.91	0.36	1.86	0.92	0.34	1.47	0.85	0.27
	[0.96,3.51]	[0.26,3.25]	[0.02,6.17]	[0.94,3.66]	[0.26,3.28]	[0.02,5.01]	[0.75,2.90]	[0.22,3.30]	[0.03,2.80]
Male X Chg. married	0.95	0.94	0.59*	0.91	0.85	0.58*	0.87	0.95	0.56
	[0.65,1.39]	[0.48,1.83]	[0.35,0.99]	[0.61,1.34]	[0.41,1.76]	[0.34,0.98]	[0.54,1.40]	[0.45,2.02]	[0.32,1.00]
Male X Chg. num. children	0.56	1.95	3.36	0.54	1.84	3.47	0.92	1.79	5.24
	[0.19,1.70]	[0.31,12.39]	[0.21,54.00]	[0.18,1.60]	[0.30,11.33]	[0.26,46.80]	[0.29,2.90]	[0.24,13.22]	[0.51,53.59]
US citizen	2.21**	2.14	1.65	2.26**	2.11	1.66	2.49**	2.08	1.73
	[1.25,3.90]	[0.75,6.10]	[0.83,3.28]	[1.29,3.97]	[0.71,6.25]	[0.82,3.36]	[1.41,4.39]	[0.52,8.40]	[0.78,3.81]
Parent is academic	0.94	0.81	1.14	0.93	0.81	1.13	1.01	0.93	1.17
	[0.62,1.44]	[0.33,2.00]	[0.62,2.10]	[0.60,1.43]	[0.33,1.97]	[0.61,2.08]	[0.63,1.62]	[0.32,2.66]	[0.63,2.17]
Started PhD in 2009	1.15	0.74	0.60	1.11	0.75	0.60	0.97	0.68	0.48*
	[0.77,1.72]	[0.41,1.36]	[0.34,1.06]	[0.72,1.71]	[0.40,1.38]	[0.34,1.04]	[0.55,1.71]	[0.35,1.33]	[0.26,0.88]
Race fixed effects	Incl.	Incl.	Incl.	Incl.	Incl.	Incl.	Incl.	Incl.	Incl.
Field fixed effects	Incl.	Incl.	Incl.	Incl.	Incl.	Incl.	Incl.	Incl.	Incl.
University fixed effects	Incl.	Incl.	Incl.	Incl.	Incl.	Incl.	Incl.	Incl.	Incl.
Constant	0.21***	0.00***	0.13**	0.17***	0.00***	0.15**	0.11***	0.00***	0.14*
	[0.09,0.49]	[0.00,0.00]	[0.04,0.47]	[0.07,0.42]	[0.00,0.00]	[0.04,0.59]	[0.03,0.36]	[0.00,0.00]	[0.03,0.63]
Log pseudolikelihood	-778.17			-773.63			-692.11		
Obs.	825			825			825		

Relative risk ratios reported. Standard errors clustered by university; 95% confidence intervals of relative risk ratios in brackets;

*** p < 0.001,

** p<0.01,

* p < 0.05.

Models 1–4 in Table 6 use the two waves of the survey separately to provide insights into the relationships between predictors and the *levels* of academic career interest at a given point in time. Models 1 and 2 use data from 2010 and Models 2 and 4 use data from 2013. We use as dependent variable the original 5-point measures of the attractiveness of the faculty research career and estimate models using ordered logit regression. As noted earlier, the most interesting aspect of these regressions is that they allow us to compare the coefficients of independent variables between the two time periods. Focusing on variables that are significant in at least one of the models, we find that the coefficients of the interest in basic research and of the importance of freedom to choose research projects are remarkably similar in the two waves (Chi^2^(1) = 0.43, p = 0.51 and Chi^2^(1) = 0.09, p = 0.76, respectively). Although the coefficients of the interest in commercialization and of the importance of salary change from insignificant in 2010 to significant in 2013, the point estimates are quite similar and the coefficients are not significantly different between the two time periods (Chi^2^(1) = 0.62, p = 0.43 and Chi^2^(1) = 3.07, p = 0.08, respectively). The positive coefficient of subjective ability is significantly larger in 2013 than in 2010 (1.48 vs. 1.24; Chi^2^(1) = 4.81, p<0.05).

**Table 6:** Ordered logit regressions predicting levels of academic interest early and later in the PhD program.

*Method*	Ordered Logit			
*Description*	Early in PhD (2010)		Later in PhD (2013)	
*Dependent variable*	Attractiveness of academic research career			
	(5-point Likert scale)			
*Model*	(1)	(2)	(3)	(4)
***Labor market expectations***				
Availability of faculty positions	1.40	0.85	1.02	0.80
	[0.73,2.67]	[0.44,1.65]	[0.52,2.00]	[0.36,1.75]
Availability of industry positions	0.84	1.00	0.65	0.64
	[0.38,1.86]	[0.38,2.69]	[0.36,1.19]	[0.31,1.34]
Number of years of postdoc	1.08	1.07	0.90	0.93
	[0.95,1.22]	[0.92,1.24]	[0.79,1.03]	[0.80,1.07]
Availability of research funding	1.03	1.10	1.32**	1.12
	[0.89,1.19]	[0.94,1.28]	[1.08,1.61]	[0.96,1.32]
***Work preferences***				
Basic research work activities		2.68***		2.46***
		[2.15,3.35]		[2.04,2.96]
Applied research work activities		1.02		1.20
		[0.69,1.51]		[0.95,1.51]
Commercialization work activities		0.88		0.81**
		[0.76,1.02]		[0.70,0.94]
Financial income		0.89		0.67**
		[0.71,1.12]		[0.51,0.88]
Freedom to choose projects		2.12***		2.24***
		[1.62,2.79]		[1.83,2.74]
***Ability***				
Self-perceived ability		1.24***		1.48***
		[1.13,1.37]		[1.31,1.66]
Number of publications		1.04		1.03
		[0.94,1.15]		[0.98,1.09]
***Control variables***				
Department NRC ranking (2010)	1.00	1.00	1.01	1.01
	[0.99,1.01]	[0.98,1.01]	[0.99,1.02]	[0.99,1.02]
Thought about career	1.09	0.94	1.14	0.87
	[0.92,1.28]	[0.81,1.10]	[0.92,1.40]	[0.68,1.12]
Male	1.93***	1.66**	1.13	1.22
	[1.45,2.58]	[1.19,2.31]	[0.71,1.79]	[0.71,2.10]
Married	2.04**	1.73*	0.76	0.62*
	[1.31,3.17]	[1.03,2.92]	[0.52,1.11]	[0.41,0.95]
Number of children	0.62	0.62	0.86	1.02
	[0.37,1.04]	[0.34,1.10]	[0.44,1.70]	[0.57,1.84]
Male X Married	0.48*	0.70	1.60*	1.74
	[0.25,0.94]	[0.34,1.43]	[1.00,2.56]	[0.94,3.21]
Male X Num. children	1.59	1.30	1.50	1.16
	[0.74,3.42]	[0.67,2.49]	[0.73,3.09]	[0.61,2.22]
US citizen	0.66*	0.47***	0.71	0.44***
	[0.46,0.94]	[0.30,0.73]	[0.48,1.04]	[0.29,0.67]
Parent is academic	0.93	0.85	1.09	1.06
	[0.63,1.36]	[0.57,1.27]	[0.86,1.37]	[0.81,1.41]
Started PhD in 2009	1.42*	1.32	0.95	0.94
	[1.06,1.89]	[0.95,1.82]	[0.68,1.33]	[0.65,1.36]
Race fixed effects	Incl.	Incl.	Incl.	Incl.
Field fixed effects	Incl.	Incl.	Incl.	Incl.
University fixed effects	Incl.	Incl.	Incl.	Incl.
Log pseudolikelihood	-948.94	-823.63	-1170.2	-945.81
Obs.	825	825	825	825

Odds ratios reported. Standard errors clustered by university; 95% confidence intervals of odds ratios in brackets;

*** p < 0.001,

** p<0.01,

* p < 0.05.

We note that publications have no significant coefficients, and per the results in Table 5 changes in publications also did not have an effect. This may reflect that any effect of publications is mediated by students’ self-perceived ability, which ultimately shapes students’ career preferences. To examine this possibility, we re-estimated key models including publications but excluding subjective ability. We find no significant coefficient in the multinomial logit regressions or in the 2010 ordered logit regressions. However, publications are highly significant in the 2013 ordered logit (odds ratio 1.08, p<0.01). This finding suggests that it is primarily self-perceived ability that influences students’ career interests, although objective measures may gain greater relevance later in the PhD program, perhaps because they are a more reliable proxy for ability than earlier in the PhD program.

Taken together, these results suggest that the predictors of career preferences are similar in both time periods, but that ability is more important closer to graduation. The latter observation may reflect that students gain a clearer understanding of the role of ability in academic success and re-evaluate the attractiveness of the faculty career in light of their own chances of performing well.

Some of the control variables also show interesting results. First, we asked students at both periods of time to what extent they had thought about their future careers. Model 3 in Table 5 shows that students who increased how much they thought about their careers were more likely to lose interest in academia. Second, the gender dummy and its interactions show that unmarried men find academia significantly more attractive than do unmarried women early in the PhD program (no significant difference between married men and women). Three years later, we find no gender difference in the attractiveness of academia among unmarried individuals but married women find academia significantly less attractive than do married men (Table 6). Finally, U.S. citizen PhD students rate academic careers significantly less attractive than foreign PhD students in both waves of the survey and they are significantly more likely to lose interest over the course of the program. These results for gender and citizenship are largely consistent with the descriptive statistics shown in Table 2, but further research is needed to examine the underlying reasons for the observed differences.

#### Auxiliary analyses

We perform three auxiliary analyses. First, recall that we found no significant association between changes in academic career interest and changes in labor market expectations, suggesting that students who lose interest in academia do not do so because their labor market expectations changed more than those of students who remain interested in academia. However, it could be that the same change in labor market expectations triggered a change in career preferences for some students but not others. In particular, students “at the margin” may respond to changed market expectations while those strongly committed to academia may not. To examine this possibility, we focus on students who had an interest in academia early in their PhD in 2010 and distinguish between those who were interested (“extremely attractive”) and those who were marginally interested (“attractive”). As expected, nearly 40% of PhD students who are at the margin lose interest between 2010 and 2013 compared to 22% of PhD students who were highly interested. We then estimate for each subsample a logit regression predicting whether a respondent loses interest in the academic career. Results in Models 1 and 2 of Table 7 show that labor market expectations have no relationship with changes in career preferences in either sample.

**Table 7:** Auxiliary analyses.

*Method*	Logit		Ordered Logit			
*Dependent variable*	Lose interest in academic research career			Change in attractiveness of academic research career		
	(relative to remain interested)					
*Sample*	Highly interested (acad. = 5)	Marginally interested (acad. = 4)	All fields	Life sciences	Physics	Engineering
*Model*	(1)	(2)	(3)	(4)	(5)	(6)
***Labor market expectations***						
Chg. availability of faculty positions	0.53	0.46	1.01	1.20	0.71	1.63
	[0.11,2.60]	[0.11,1.97]	[0.58,1.77]	[0.37,3.83]	[0.15,3.28]	[0.32,8.29]
Chg. availability of industry positions	2.71	1.72	0.80	1.90	0.61	0.25
	[0.39,18.90]	[0.43,6.91]	[0.53,1.18]	[0.70,5.10]	[0.18,2.07]	[0.05,1.17]
Chg. number of years of postdoc	1.25	0.88	0.98	1.14	0.91	1.10
	[0.72,2.17]	[0.69,1.12]	[0.87,1.10]	[0.93,1.40]	[0.68,1.20]	[0.91,1.33]
Chg. availability of research funding	1.08	1.05	1.11	1.04	1.03	1.18
	[0.66,1.77]	[0.80,1.39]	[0.96,1.29]	[0.75,1.43]	[0.61,1.75]	[0.75,1.85]
***Work preferences***						
Chg. basic research work activities	0.28***	0.40***	1.89***	2.28***	3.19**	1.75*
	[0.14,0.58]	[0.24,0.67]	[1.61,2.22]	[1.62,3.20]	[1.38,7.41]	[1.09,2.82]
Chg. applied research work activities	0.74	0.71	1.07	0.90	0.83	1.20
	[0.36,1.50]	[0.46,1.11]	[0.91,1.27]	[0.69,1.17]	[0.31,2.20]	[0.72,1.98]
Chg. commercialization work activities	1.12	1.28	0.84**	1.02	0.80	0.60***
	[0.72,1.73]	[0.92,1.78]	[0.74,0.95]	[0.75,1.39]	[0.57,1.13]	[0.45,0.80]
Chg. financial income	1.33	0.90	1.01	1.06	1.25	0.75
	[0.71,2.48]	[0.61,1.34]	[0.84,1.23]	[0.76,1.47]	[0.55,2.84]	[0.50,1.13]
Chg. freedom to choose projects	0.58	0.65*	1.67***	1.75***	1.02	2.02***
	[0.27,1.25]	[0.46,0.91]	[1.41,1.97]	[1.36,2.25]	[0.65,1.59]	[1.34,3.04]
***Ability***						
Chg. self-perceived ability	0.79	0.75**	1.28***	1.51***	1.31**	1.35*
	[0.62,1.02]	[0.60,0.93]	[1.19,1.39]	[1.23,1.84]	[1.08,1.59]	[1.06,1.72]
Chg. number of publications	1.15	0.90	1.05	1.06	1.02	1.09
	[0.80,1.64]	[0.76,1.06]	[0.98,1.13]	[0.92,1.22]	[0.74,1.40]	[0.90,1.31]
***Control variables***						
Department NRC ranking (2010)	1.00	0.99	1.01	1.02	0.99	1.02
	[0.94,1.07]	[0.95,1.02]	[0.99,1.02]	[0.99,1.04]	[0.89,1.10]	[0.98,1.06]
Chg. thought about career	1.04	1.42*	0.86*	0.84	0.68	0.80
	[0.61,1.77]	[1.04,1.93]	[0.76,0.97]	[0.64,1.11]	[0.35,1.31]	[0.59,1.07]
Other individual control variables	Incl.	Incl.	Incl.	Incl.	Incl.	Incl.
Race fixed effects	Incl.	Incl.	Incl.	Incl.	Incl.	Incl.
Field fixed effects	Incl.	Incl.	Incl.	Incl.	Incl.	Incl.
University fixed effects	Incl.	Incl.	Incl.	Incl.	Incl.	Incl.
Constant	0.02*	0.22				
	[0.00,0.68]	[0.03,1.34]				
Log pseudolikelihood	-85.27	-160.7	-1132.58	-369.24	-172.67	-234.31
Obs.	240	335	825	302	145	192

Odds ratios reported. Standard errors clustered by university; 95% confidence intervals of odds ratios in brackets;

*** p < 0.001,

** p<0.01,

* p < 0.05.

Second, we simplify the analysis by using a change score as the dependent variable, computed as the difference between respondents’ interest in a faculty career early (2010) and later (2013) in the PhD program. This variable ranges from -4 to 3, with a mean of -0.55 and 43% of respondents reporting no change (i.e., zero). Compared to our dichotomized primary measure, this variable reflects the extent to which career preferences change over the whole range, without relying on a qualitative threshold. At the same time, this measure does not distinguish between individuals who lose interest from a high starting level (e.g., from 5 to 4, for a change of -1) and those who lose interest from a low starting level (e.g., from 2 to 1, also for a change of -1). We regress this change score using an ordered logit regression. Model 3 in Table 7 uses the full sample and shows that the results are largely consistent with our main analysis: We find no significant coefficients of labor market expectations, but significant positive coefficients of changes in respondents’ preferences for basic research and freedom, as well as changes in subjective ability. Moreover, we find that changes in the preference for commercialization activities are negatively related with changes in academic interest.

Finally, given that our sample size is too small to estimate multinomial regressions separately by field, we instead estimate models using the change score for our three largest fields: life sciences, physics, and engineering. The results (Table 7, Models 4–6) show no significant coefficients of labor market expectations. Changes in the interest in basic research are positively related to changes in academic career interest in all three fields, although the coefficients are larger in the sciences than in engineering. Among engineering PhD students, changes in the interest in commercial activities have a strong and significant negative relationship with changes in academic career interests. Thus, changes in preferences for different work activities appear to play a role in all three fields, although the particular activities that matter may differ depending on the dominant kind of work done in these fields [31]. We also find that changes in the importance of research freedom are positively related to changes in academic career interests in the life sciences and in engineering, but not in physics, while changes in subjective ability are positively related to changes in academic career interests in all fields. Given the small sample size, these analyses should be interpreted with caution. However, they point toward the value of future work that more explicitly considers field differences in the dynamics of students’ career interests.

### Limitations

Before we turn to implications, it is important to highlight a number of limitations and opportunities for future research. First, although we explored a number of market and non-market reasons that may underlie changes in students’ interests in the faculty career, there may be other reasons that we were not able to examine. Relatedly, our focus was on changes in students’ academic career interests and future research is needed to study whether and why students also experience changes in their interests in non-academic careers. Second, we described some differences in the dynamics of career preferences by field and demographic characteristics. Unfortunately, the sample is not large enough to perform a more systematic analysis of potential drivers of changes in career preferences for different sub-populations. Third, the use of multiple survey questions for a given construct can increase reliability and researchers’ ability to detect relationships among variables. Although the use of single item measures allowed us to reduce respondent burden and to explore a broad range of factors, future work should examine key relationships using multi-item measures. Finally, our data do not speak to the dynamics of career preferences outside of science and engineering fields.

Although we are not aware of other longitudinal data on PhD students’ career preferences, a survey sponsored by the Pew Charitable Trust in 1999 covered a broader range of fields and included a question asking PhD students retrospectively whether their interest in becoming a professor in a college or university had changed since the start of the program [16]. The Pew survey showed that the shares of students who reported a decreased interest in this career was considerably larger in the biological sciences and the physical sciences (43% and 40%, respectively) than in the humanities and the social sciences (29% and 32%). Although major differences in question formats and samples do not allow a quantitative comparison with our data, the Pew study reinforces some important points: First, changes in career preferences over the course of the PhD training are considerable, and there is strong evidence in particular for a decline in students’ interest in the academic career path. Second, while such changes likely occur in all fields, they appear most pronounced in the physical and biological sciences.

## Discussion

We reported a range of complementary analyses that yield a number of key insights. We now summarize these insights and discuss important implications. First, although labor market conditions almost certainly prevent some graduates who are interested in an academic career from obtaining a faculty position, we find that a substantial share of PhD students lose interest in an academic research career for reasons other than labor market conditions. As such, efforts to understand students’ career paths should consider the diversity in career goals and a broad range of factors that shape these goals. In particular, comparisons of the number of graduates with the number of available faculty positions [2, 7, 8] likely overstate the number of PhDs who aspire to a faculty career, thereby exaggerating imbalances in academic labor markets (see also [4]). This insight provides urgency to the National Academies’ recent call for better data on students’ career preferences [6] and we present a measure that may be useful in such data collection efforts.

Second, there is considerable heterogeneity in the degree to which career preferences change. While many students remain highly interested in an academic research career, others report a significant decrease in their interest in academia. The large share of students who remain interested alleviates concerns about a potential “drying up” of the pipeline of highly trained scientists pursuing academic careers. While the declining interest in academia among other students may concern observers who believe that all PhDs should aspire to a faculty career, these changes may also be seen as positive to the extent that they result in a better alignment between students’ career preferences and the careers they ultimately enter.

Third, a significant share of advanced students– 40% in our study—are not interested in pursuing an academic career. Given that many students report a lack of information about non-academic career options [15] this finding suggest that better information about a variety of career pathways earlier in the PhD may be beneficial [6, 32]. Workshops and information sessions are offered by many institutions [33] but may have a limited ability to truly convey what it means to work in other sectors. Experiential approaches such as internships may be more effective by allowing students to experience non-academic careers first-hand. Moreover, there is the concern that career exploration may be hindered by a lack of support from advisors, who tend to strongly encourage the traditional academic career path [4, 34]. As such, allowing students the time to explore different career options and creating an open culture that acknowledges changing preferences and that values non-academic career paths may be important complements to offering richer information [7, 35]. Students, in turn, should begin to consider their careers early on and take advantage of the career exploration opportunities provided by their advisors and programs.

It is well recognized that graduate schools need to prepare PhD students for a variety of academic and non-academic careers [6]. Several innovative initiatives—such as NIH’s BEST program—are important steps towards this goal. Our results suggest that such initiatives need to take a dynamic perspective to accommodate changing career preferences over the course of graduate training. In particular, if students enter PhD programs aspiring to faculty careers, they are unlikely to take advantage of opportunities to explore non-academic options right away. In addition to encouraging students to explore different career options and interests, programs should thus provide students with the flexibility to adjust and modify program components as their career goals change.

Finally, future research is needed on whether and how some of the learning that appears to underlie the observed changes in career preferences can be accelerated or even moved *prior to* students’ enrolling in a PhD program. More explicit assessments of their own interests and abilities, as well as more realistic evaluations of career options may lead some individuals to realize that pursuing a faculty career, and a PhD, is not the best way forward for them. This may allow individuals to take advantage of a growing range of alternative educational options, such as professional science master’s programs, and ultimately result in faster career progress and more satisfying long-term career outcomes.

## Supporting information

S1 Table Variables and measures. (DOCX)

S1 Text Survey questionnaire. (DOCX)
