# Supplementary material for: The declining interest in an academic career
Source: PLoS One. 2017 Sep 18;12(9):e0184130. doi: 10.1371/journal.pone.0184130 (PMC5602526; doi:10.1371/journal.pone.0184130)
Supplement: S1 Table — (DOCX) [file pone.0184130.s001.docx]

**S1 Table. Variables and measures**

| ***Variable*** | **Survey question** | **Response scale** | **Mean (2010)** | **Mean (2013)** |
| --- | --- | --- | --- | --- |
| ***Career Interests*** |  |  |  |  |
| Attractiveness of research faculty career | “Putting job availability aside, how attractive or unattractive do you personally find a university faculty career with an emphasis on research or development?” | 5-point scale ranging from “Extremely unattractive" to “Extremely attractive" | 4.04 | 3.49 |
| ***Labor market expectations*** |  |  |  |  |
| Availability of faculty positions | “What do you think is the probability that a PhD in your field can find a university faculty position with an emphasis on research or development after graduation (and any potential postdocs)” | 0-100% | 0.48 | 0.32 |
| Availability of industrial R&D positions | “What do you think is the probability that a PhD in your field can find an established firm job with an emphasis on research or development after graduation (and any potential postdocs)” | 0-100% | 0.64 | 0.59 |
| Number of years of postdoc | “How many years of postdoc experience do you think are required on average to obtain a university faculty position with an emphasis on research or development in your field?” | 0 years - 5 or more years | 2.74 | 3.06 |
| Availability of research funding | "To what extent do you think research funding is available to university faculty?" | 5-point scale ranging from “Extremely low" to “Extremely high" | 3.22 | 2.74 |
| ***Work preferences*** |  |  |  |  |
| Basic research work activities | "When thinking about the future, how interesting would you find working on research that contributes fundamental insights or theories (basic research)?" | 5-point scale ranging from “Extremely uninteresting” to “Extremely interesting" | 4.19 | 3.88 |
| Applied research work activities | "When thinking about the future, how interesting would you find working on research that creates knowledge to solve practical problems (applied research)?" | 5-point scale ranging from “Extremely uninteresting” to “Extremely interesting" | 4.34 | 4.26 |
| Commercialization work activities | "When thinking about the future, how interesting would you find commercializing research results into products and services?" | 5-point scale ranging from “Extremely uninteresting” to “Extremely interesting" | 3.21 | 3.26 |
| Financial income | "When thinking about an ideal job, how important to you is financial income (e.g., salary, bonus)?" | 5-point scale ranging from “Not at all important” to “Extremely important” | 3.90 | 3.94 |
| Freedom to choose work projects | "When thinking about an ideal job, how important to you is freedom to choose research projects?" | 5-point scale ranging from “Not at all important” to “Extremely important” | 4.18 | 3.90 |
| ***Individual Characteristics*** |  |  |  |  |
| Number of publications | "How many articles published or accepted in peer-reviewed journals list you as an author?" | Multiple choice ranging from 0 to 5+ | 0.87 | 2.52 |
| Self-perceived ability | "How would you rate your research ability relative to your peers in your area of specialization?" | Slider bar ranging from "Among the least skilled" to "Among the most skilled" | 6.17 | 6.64 |
| Thought about career | "Generally speaking, to what extent have you thought about your future career plans?" | 5-point scale ranging from "Not at all" to "Great extent" | 3.42 | 3.97 |
| Male | "What is your gender?" | 1 if male, 0 if female | 0.59 | 0.59 |
| U.S. citizen | "Are you a U.S. citizen?" | 1 if U.S. citizen, 0 otherwise | 0.74 | 0.76 |
| Married | "What is your marital status?" | 1 if married, 0 otherwise | 0.31 | 0.55 |
| Number of children | "How many children do you have under the age of 18?" | Count of number of children | 0.07 | 0.15 |
| Started PhD in 2009 | Year started PhD | 1 If started PhD in 2009, 0 if started PhD in 2008 | 0.38 | 0.38 |
| Race fixed effects | “Which of the following best describes your ethnic/racial background?” | African-American, Asian, Hispanic or Latino, White, Other |  |  |
| Field fixed effects | Respondent reported field of study | Respondents used a dropdown menu to report their field/area of specialization |  |  |
| University fixed effects | 39 university dummy variables |  |  |  |
