# Supplementary material for: The declining interest in an academic career
Source: PLoS One. 2017 Sep 18;12(9):e0184130. doi: 10.1371/journal.pone.0184130 (PMC5602526; doi:10.1371/journal.pone.0184130)
Supplement: S1 Text — (DOCX) [file pone.0184130.s002.docx]

**S1 Text: Survey questionnaire**

**Career interests**

**Q1: Putting job availability aside, how attractive do you personally find each of the following careers?**

|  | Extremely unattractive | Unattractive | Neither attractive nor unattractive | Attractive | Extremely attractive |
| --- | --- | --- | --- | --- | --- |
| University faculty with an emphasis on research or development |  |  |  |  |  |

**Labor market expectations**

**Q2: What do you think is the probability that a PhD in your field can find the following positions after graduation (and any potential post-docs):**

(*SLIDER BARS form 0-100:* Low probability of finding a job, Medium probability of finding a job, High probability of finding a job)

1. University faculty with an emphasis on research or development
2. Established firm job with an emphasis on research or development

**Q3: How many years of postdoc experience do you think are required on average to obtain each of the following positions in your field?**

(Multiple choice response: 0, 1, 2, 3, 4, 5, 6, 7 or more)

**Q4: To what extent do you think the following job attributes are available to PhD researchers in your field in a research university and an established firm, respectively?**

|  | Extremely low | Moderately low | Moderate | Moderately high | Extremely high |
| --- | --- | --- | --- | --- | --- |
| Availability of research funds |  |  |  |  |  |

**Preferences for work activities and job attributes**

**When thinking about the future, how interesting would you find the following kinds of work?**

|  | Not at all interesting | Uninteresting | Neither interesting nor uninteresting | Interesting | Extremely interesting |
| --- | --- | --- | --- | --- | --- |
| Research that contributes fundamental insights or theories (basic research) |  |  |  |  |  |
| Research that creates knowledge to solve practical problems (applied research) |  |  |  |  |  |
| Using knowledge to develop materials, devices, or software (development) |  |  |  |  |  |
| Commercializing research results into products or services |  |  |  |  |  |

**When thinking about an ideal job, how important is each of the following factors to you?**

| (*random order*) | Not at all important | Unimportant | Neither important nor unimportant | Important | Extremely important |
| --- | --- | --- | --- | --- | --- |
| Freedom to choose R&D projects |  |  |  |  |  |
| Financial income (e.g., salary, bonus) |  |  |  |  |  |

**Individual characteristics**

**How many of each of the following list you as an author or inventor? Please select 0 if none.**

|  | 0 | 1 | 2 | 3 | 4 | 5 | 6 | 7 | 8 or more |
| --- | --- | --- | --- | --- | --- | --- | --- | --- | --- |
| Articles published or accepted in peer-reviewed journals |  |  |  |  |  |  |  |  |  |

**How would you rate your research ability relative to your peers in your area of specialization? Please use your mouse to slide the bar to select the most appropriate response.**

*SLIDER*: Among the least skilled, Below average, Average, Above average, Among the most skilled

**
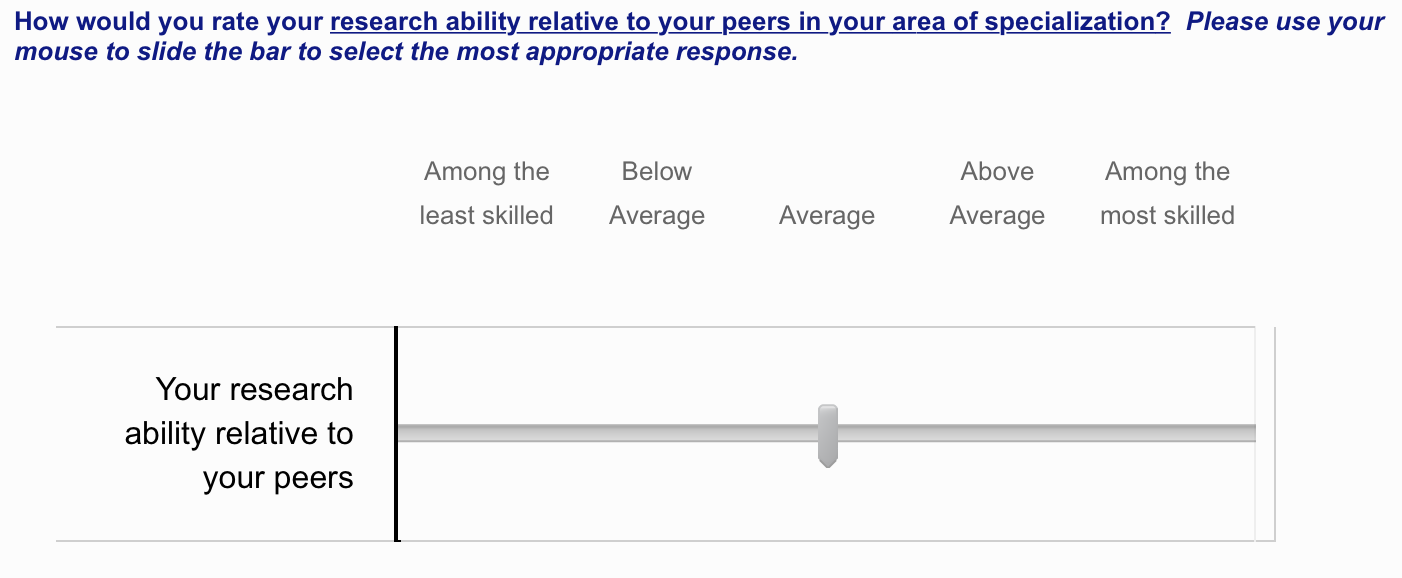
**

**
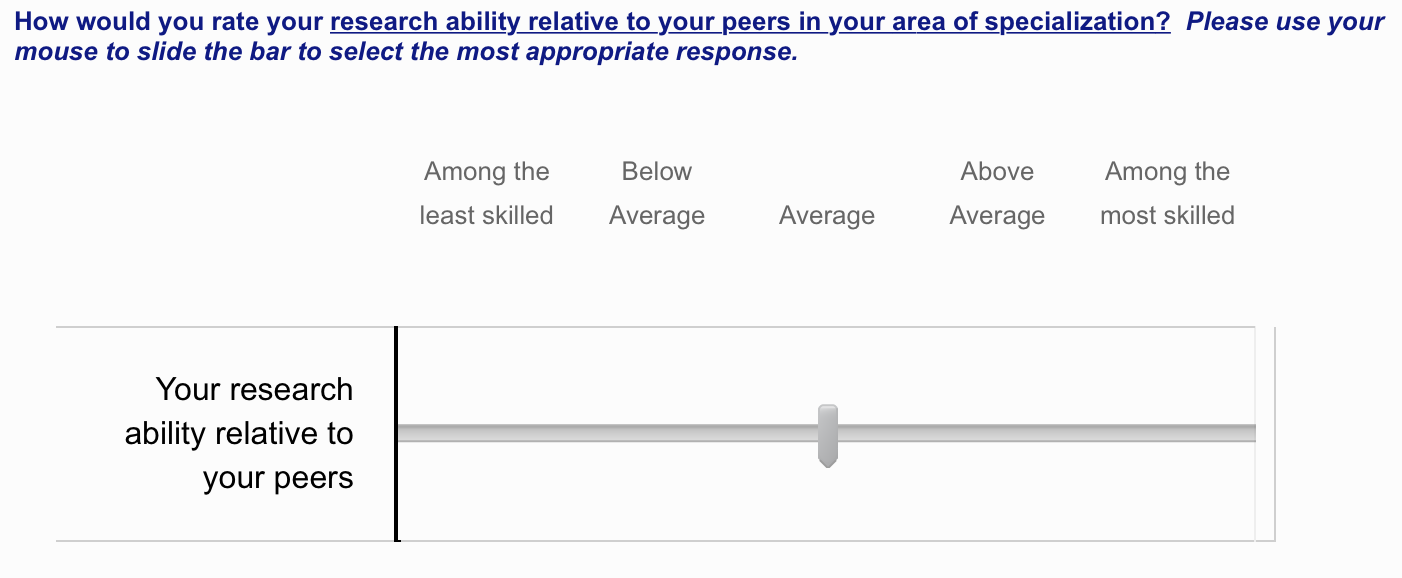
**

**Generally speaking, to what extent have you thought about your future career plans?**

1. Not at all
2. Small extent
3. Some extent
4. Large extent
5. Great extent
